# Associations Between Local Health Department Expenditures on Foundational Capabilities and PHAB Accreditation Standards Scores

**DOI:** 10.3389/fpubh.2022.861587

**Published:** 2022-05-25

**Authors:** Oluwatosin O. Dada, Betty Bekemeier, Abraham Flaxman, A. B. de Castro

**Affiliations:** ^1^Department of Child, Family, and Population Health Nursing, School of Nursing, University of Washington, Seattle, WA, United States; ^2^Department of Health Metrics Sciences, School of Medicine, University of Washington, Seattle, WA, United States; ^3^Department of Global Health, School of Public Health, University of Washington, Seattle, WA, United States

**Keywords:** Local Health Departments, Foundational Capabilities, Resource Allocation, Public Health Accreditation, Accountability and Performance Management, Population Health

## Abstract

**Context:**

Foundational Capabilities (FC) are the public health (PH) infrastructure areas that are essential for local health departments (LHDs) to support a “minimum package” of programs and services that promote population health. Despite being a critical component of LHD programs, FC are chronically underfunded, and studies specific to the relationship between LHD FC expenditures and their performance—the LHDs' ability to provide essential PH programs and services to their community—have not been previously reported. Public Health Accreditation Board (PHAB) accreditation is a nationally recognized accreditation program for PH agencies. PHAB accreditation assesses LHDs' performance against sets of standards that are based on the 10 essential PH services. Alignment between FC and the PHAB standards presents a means for assessing LHD FC expenditures relative to their performance in PHAB accreditation standards.

**Objectives:**

We examined the association between LHD total FC expenditures, as well as FC funding allocation patterns, and performance score on selected PHAB accreditation standards.

**Methods:**

We used Bayesian regression methods to estimate the coefficients for the aggregate performance score, and performance scores on individual PHAB standards.

**Results:**

Analyses showed that a dollar increase in total FC expenditures is associated with a 0.2% increase in the aggregate performance score in selected PHAB standards as well as the performance score on most of the standards examined. LHDs that allocated FC budgets more evenly across FC programs were found to be more likely to have higher scores.

**Conclusions:**

Investment in FC could improve LHD performance scores in PHAB accreditation standards and support LHDs' capability for improving community health outcomes. Allocating available FC resources across the various FC programs could support better LHD performance, as indicated by accreditation scores. This study contributes to advancing the understanding of public health finances in relation to performance and could help guide effective LHD resource allocation.

## Background

Local health departments (LHDs) play key roles in promoting and protecting local communities' health (1, 2), through provision of essential population-based programs and services in infectious disease control, chronic disease and injury prevention, environmental public health, maternal and child health, access to and linkage with clinical care, and other major programs (3, 4). LHDs are crucial elements of the public health (PH) system and are expected to equitably maintain PH programs and services across communities in the United States. However, differences in LHD resources and cross-cutting infrastructure capabilities (5–9) result in variability in their performance—the ability to provide essential PH programs and services to their community (10). Variability in LHD performance creates unequal community access to public health services across jurisdictions (5, 6, 11–15). Thus, PH leaders seek opportunities to advance LHDs' performance through improved resources and infrastructure capabilities (8, 9, 16–18).

In 2012, the National Academy of Medicine (NAM) called for LHDs to maintain a “minimum package” of programs to support effective responses to public health crises and to assure population health is promoted equitably (7, 16, 19, 20). NAM's recommendation led to the establishment of the Foundational Public Health Services (FPHS) framework. The framework established a minimum set of Foundational Capabilities (FC) that LHDs should maintain in every jurisdiction to support a core set of Foundational Services (FS) to assure basic health promotion and protection in all communities (7, 20). These cross-cutting infrastructure capabilities include Assessment and Surveillance, Emergency Preparedness and Response, Policy Development and Support, Communications, Community Partnerships Development, Organizational Administrative Competencies, and Accountability and Performance Management (20). There is growing interest among PH leaders to understand how investments in FC relate to performance in PH functions, so that PH systems can be most responsive to local needs (9, 16, 21, 22). FC are considered the “building blocks” for LHDs' services such as maternal, child, and family health; immunization programs; and communicable disease control (8, 20).

Several studies have reported associations between LHD performance and PH funding (23, 24), PH expenditures (25), and PH infrastructure (23, 24). However, studies on the association between FC and performance are limited. An earlier study by Scutchfield et al. (24) showed that higher spending in organizational competency, a sub-component of FC, relates to significantly higher performance in nine out of the 10 essential PH services. Mays et al. (26). also found a positive association between PH expenditures on programs that relate to PH agencies' capabilities and agency performance The framework for these previously reported studies from the early 2000's was based on performance information collected through survey-driven, self-reported evaluation that focused on individual LHDs. A number of these studies noted the need for a standardized national performance assessment instrument (22, 24, 27, 28), as variations in LHDs' data structure and reporting processes often hinder comparative assessment of agency performance (16, 28–30).

Public Health Accreditation Board (PHAB) accreditation is a nationally recognized program for assessing public health agencies' capacity to deliver the 10 essential PH services (31). PHAB was launched in 2011 as a national voluntary accreditation program for PH departments with the aim of improving and protecting population health by advancing the performance and quality of the nation's PH agencies (32–37). Accreditation recognizes health departments that meet standards designed to assess agencies' readiness to respond to PH needs and facilitates continuous quality improvement and performance (36). PHAB accreditation serves as a national performance assessment for PH that had been noted as a need in previous studies (22, 24, 27, 28).

Though the establishment of FPHS and PHAB were different initiatives, they both describe core elements of PH practice that are meant to create healthy communities (38), and the alignment of these elements within them has been established. An overview of this PHAB and FPHS alignment is shown in Table 1 (38). Expenditures on FC is an indicator of the level of FC [including infrastructure areas such as Assessment and Surveillance, Emergency Preparedness and Response, Policy Development and Support (8, 9, 20)] that exist in a LHD and evidence suggests there is a link between FC expenditure and PHAB accreditation (38). A recent study by Singh et al. (9) examined variation in LHDs' spending on FC among 16 LHDs from four states, and noted that accredited LHDs tend to have higher spending on FC than those that were not accredited. In this study we describe here, rather than looking at LHDs' PHAB accreditation status (i.e., whether an LHD is accredited or not) in relation to FC expenditures, we examined LHDs' actual performance scores in PHAB accreditation, as a measure of their ability to provide essential PH services, relative to their FC expenditures.

**Table 1:** Overview of FPHS and PHAB Domain alignment.

**FPHS**	**PHAB Standards & Measures Version 1.5 Domains**											
	**1**	**2**	**3**	**4**	**5**	**6**	**7**	**8**	**9**	**10**	**11**	**12**
Assessment/surveillance												
Emergency preparedness and response												
Policy development and support												
Communications												
Community partnership development												
Organizational administrative competencies:												
- Leadership and governance												
- Health equity												
- Information technology services, including privacy and security												
- Human resources services												
- Financial management, contract, and procurement services, including facilities and operations												
- Legal services and analysis												
Accountability/performance management												
- Quality improvement												

*The FPHS column lists each activity per the national FPHS model. The PHAB Standards and Measures Version 1.5 Domains columns provide one column per PHAB domain. Where there is alignment between the FPHS activities and the PHAB domains, the cells are filled in. This excerpt was from the Public Health National Center for Innovations. https://phnci.org/uploads/resource-files/Aligning-Accreditation-and-the-Foundational-Public-Health-Capabilities-November-2018.pdf (38)*.

Studying the association between LHD performance scores in PHAB accreditation standards and their expenditures on FC could support a better understanding of LHDs' financial management strategies that maximize accreditation outcomes, and, thus, provide information that could help guide accreditation policy and improved performance for LHDs. The aims of this study were to examine the association between the amount of total FC expenditures, as well as LHD budget allocation to the seven broad FC areas, and LHD performance scores in PHAB accreditation standards.

## Methods

We used Bayesian regression methods to estimate the coefficients for the aggregate performance score, and performance scores in individual PHAB standards with respect to LHDs' expenditures on FC. LHD performance was depicted as the LHDs' performance score in PHAB Accreditation Standards since PHAB standards are indicators of the 10 essential public health services.

### Data

There were multiple data sources for this study. Accreditation scores were obtained from the dataset of 250 accredited LHDs from PHAB (36). Population size data for each LHD jurisdiction came from the 2014 Census estimate, as reported in the 2016 National Association of County and City Health Officials (NACCHO) Profile Survey. FC expenditures and FS expenditures came from the University of Washington Public Health Activities and Services Tracking (PHAST) Uniform Charts of Accounts (UCOA) dataset of detailed, standardized LHD financial data (39). The UCOA dataset included cross-sectional data of the 67 LHDs from 18 states that had chosen to participate in the UCOA and for which data were collected between 2015 and 2020. However, only 31 of these LHDs had completed PHAB accreditation at the time of this analysis. Data regarding LHD organizational structure and education level of LHD lead executives were also obtained from the 2016 NACCHO Profile Survey (1, 4); we included these data due to previous research suggesting their relationship to LHD performance and accreditation involvement (24, 40–42). All dollar amounts were converted to per capita expenditures based on each LHD jurisdiction's population size. The selected standards and FC areas alignment are described in the Supplementary Appendix 1.

#### Performance Measure: LHDs' Scores in PHAB Standards

The PHAB data categorize LHDs based on each LHD's proficiency in various accreditation standards and measures that relate to the 10 essential PH services (43). The data are organized into 12 domains, 32 standards, and over 100 measures (44). Domains are the groups of standards that relate to a broad group of public health services (20, 31, 45). Standards are the level of attainment that an LHD is expected to meet in a domain (31, 38). Measures are the metrics for evaluating proficiency in a specific standard, and, each standard contains at least two measures (31). Details of PHAB domains, standards, and measures are available elsewhere (31). In accreditation assessment, an LHD is scored by trained external reviewers in each of the PHAB measures on an ordered nominal scale of “Not Demonstrated” to “Fully Demonstrated,” with a range of four categories. For analytical purposes, we transformed the categorical scores into a numeric scale by assigning 0.25 to represent one unit on the Likert scale, with a score of 0.25 corresponding to “Not Demonstrated,” and 1.0 corresponding to “Fully Demonstrated.” We defined performance score in each standard as the average of scores for all measures in the standard. Aggregate performance score is defined as the average of performance scores in all the individual standards examined.

The PHAB standards examined in this study are a subset of those established by the Public Health National Center for Innovation as being directly aligned with FC (38). The selected standards were those that uniquely align with FC infrastructure areas and minimally overlap with other standards. An overview of the selected 19 standards, the corresponding domains, and the connection with the FC infrastructure areas is shown in Supplementary Appendix 1. We used the nomenclature we created in another study (46) to represent the domain-standard composite, where Domain 2, Standard 3 is represented as D2S3, for example.

#### Total FC Budget and FC Allocation Pattern

The UCOA data contained uniquely detailed and comparable financial data for a total of 67 LHDs whose leaders had cross-walked their financial reports into the PHAST UCOA and for which there were no missing data. From the UCOA data, we extracted LHD expenditures on FC and FS. We defined Total FC Budget as the aggregate total spending on the seven FC infrastructure areas as characterized by the FPHS framework (20). FC Allocation was the percentage of the total FC budget allocated to each FC infrastructure area. FC Allocation Pattern was then defined as the distribution of FC Allocation across the seven FC infrastructure areas. Only accredited LHDs from the UCOA dataset (*n* = 31) were used for modeling LHD performance score.

#### Other Covariates

Foundational Services budget was the total spending on the FS programs as defined by the FPHS framework (20). Education level of LHD lead executives was coded as 1—Master's degree or higher and 0—bachelor's degree or lower. Population groups 1 (>I million population size), 2 (250,000–1 million population size), and 3 (100,000–249,999 population size) were as defined in the UCOA data. Population groups 4, 5, and 6 from the UCOA data were merged, because they overlapped in the UCOA dataset, to create population group 4 (<100,000 population size). Performance cluster number was the index derived from a k-means cluster analysis of performance scores in PHAB standards and used to assign LHDs into groups relative to their performance scores (46). In the performance clustering conducted with a much larger dataset (*n* = 250) of LHDs, three groups were identified (46). In the data set for this study, each LHD inherited the cluster group from the cluster analysis done with the lager sample (*N* = 250).

### Analysis

First, we used *k*-means cluster analysis to categorize the initial 67 LHDs with UCOA data into similar groups based on their budget allocation pattern in FC program areas. We then applied the cluster indices to the accredited LHDs from each cluster group identified. The cluster group indices (FC Cluster 1, FC Cluster 2, and FC Cluster 3) for the accredited LHDs (*n* = 31) were used in modeling performance score as a function of FC Allocation pattern.

For the regression analyses, we used Bayesian methods to evaluate the association between LHD FC budget amounts (Total FC Budget) and performance scores in the PHAB accreditation standards, and between FC pattern clusters (FC Allocation) and performance scores in PHAB accreditation standards. The regression analyses were performed using the *stan_glm*() function in *rstanarm* package. Total FC Budget and FC Allocation were the primary independent variables examined separately with performance score in the PHAB standards. For both models, we used standard student-t (*df* = 7) prior with a mean of 0. The model parameters were estimated for the performance score in each of the selected 19 PHAB standards, and their average. Percent difference in performance score (relative to a unit change in FC expenditures) was calculated by transforming the loglinear coefficients to a linear scale.

The model for examining associations between Total FC Budget and performance scores in PHAB accreditation standards included FS expenditure, education level of the LHD lead executive, and performance cluster as covariates to control for their potential effects. The model for examining associations between FC Allocation Pattern and performance scores in PHAB accreditation standards included Total FC Budget and education level of the LHD lead executive as covariates to control for their potential effects. The FC Allocation model did not control for FS expenditures because, once a total FC budget has been determined, total FS budget is unlikely to impact decisions regarding the distribution of FC budget to various FC infrastructure areas.

#### Model Variable Selection

To determine the model specification for examining the association between Total FC Budget (primary independent variable) and PHAB accreditation score (dependent variable), we used a fixed effect loglinear model using *glm*() for different model specifications. We used the Bayesian information criterion (BIC) and Akaike information criterion (AIC) as diagnostics for variable selection. Table 2 shows the list of models tested and the corresponding diagnostic values. Model 5 had the lowest BIC and AIC, suggesting the best model fit. All data treatment and statistical analyses were performed in R Software package version 4.0.2.

**Table 2:** Model variable selection with BIC and AIC diagnostics.

**Model**	**Variables**	**BIC**	**AIC**
1	fcapbudget + fserbudget + exeeducode + version + authority + log(MEDHHINC) + pctLessHS + phabscorecluster	−28.36285	−42.20191
2	fcapbudget + fserbudget + exeeducode + version + log(MEDHHINC) + pctLessHS + phabscorecluster	−43.908	−58.248
3	fcapbudget + fserbudget + exeeducode + version + log(MEDHHINC) + phabscorecluster	−43.306	−56.212
4	fcapbudget + fserbudget + exeeducode + version + phabscorecluster	−44.801	−56.273
**5**	**fcapbudget** **+** **fserbudget** **+** **exeeducode** **+** **phabscorecluster**	–**48.096**	–**58.134**
6	fcapbudget + fserbudget + phabscorecluster	−46.350	−54.954
7	fcapbudget + fserbudget + exeeducode + version + authority + phabscorecluster	−32.788	−44.111
8	fcapbudget + fserbudget + authority + phabscorecluster	−36.114	−44.921
9	fcapbudget + fserbudget + version + phabscorecluster	−43.448	−53.487
10	fcapbudget + fserbudget + version + authority + phabscorecluster	−34.377	−44.442
11	fcapbudget + fserbudget + exeeducode + version + phabscorecluster	−44.801	−56.273

***Authority**: Number of budget related areas under the LBH authority.*

***CountyNum**: Number of Jurisdictions served by an LHD.*

***medhhinc**: Per Capita median household income of the jurisdiction served by an LHD (US Dollar).*

***totalPHExp**: Per Capita Total Public Health Expenditure (US Dollar).*

***ExecEdu**: Education level of LHD executive (1-Associate, 2-Bachelor, 3-Masters, 4-Doctorate).*

***Version**: PHAB Accreditation version (version 1.0 or version 1.5).*

***Kmeanscltr**: PHAB Standard Score Cluster (1—Moderate Score, 1—Low Score, 3—Low Score).*

*The dependent variable was the Average Score of the selected 19 PHAB standards*.

## Results

### Descriptives

**Figure 1:**
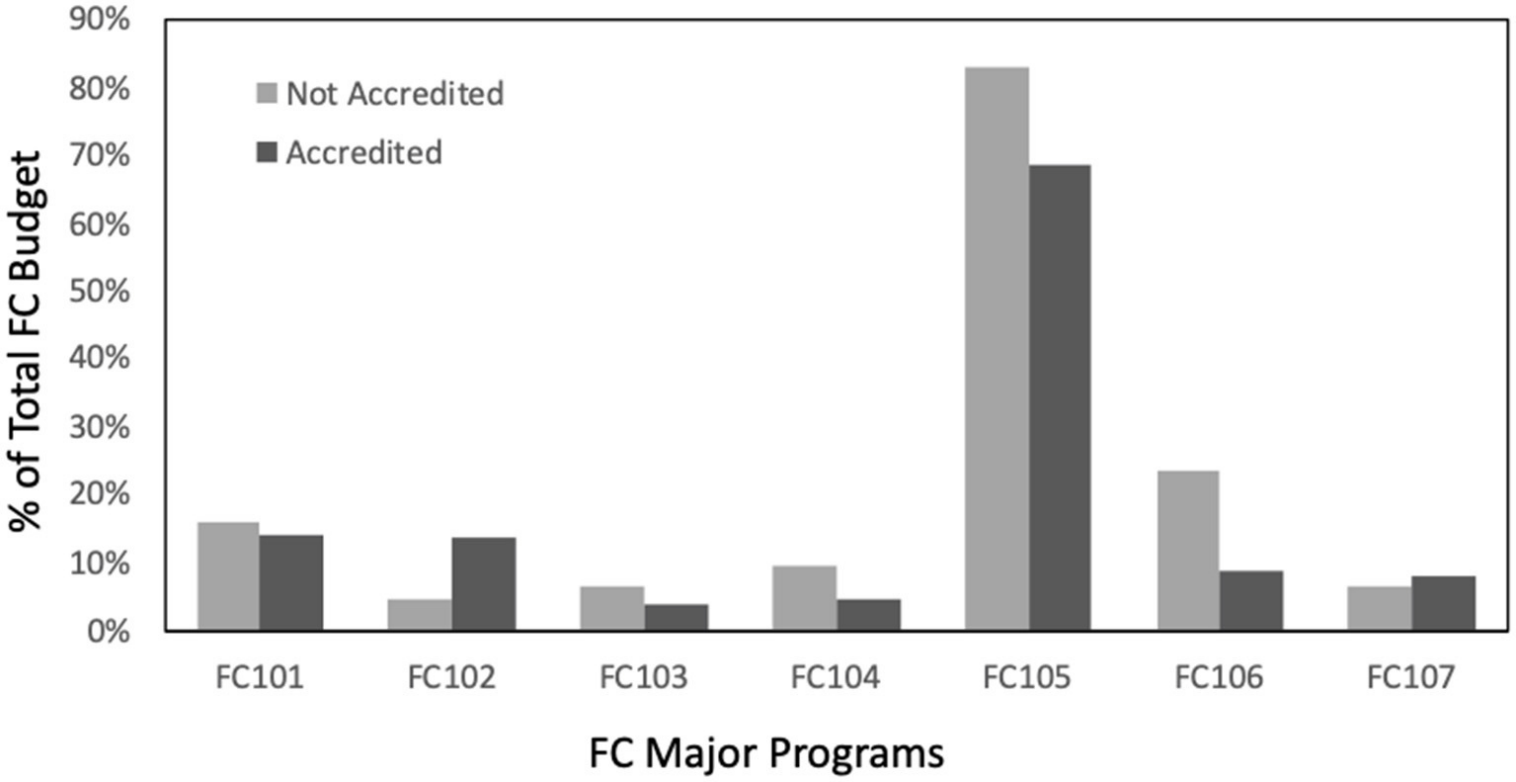
Distribution of FC expenditures relative to allocations among specific FC program areas. Fcap101: Emergency preparedness and Response, fcap102: Assessment, fcap103: Communication, fcap104: Community Partnership and Development, fcap105: Organizational Competency, fcap106: Policy Development and Support, fcap107: Other Capabilities.

There were no major differences between performance scores in each of the 19 selected PHAB standards for the sample of 31 accredited LHDs with UCOA expenditure data, when compared to the scores for the total sample of 250 LHDs in the full PHAB dataset. Similarly, there was no major difference in the average Total FC Budget and spending in each FC infrastructure area for the accredited and not-accredited LHDs in the sample. Also, the distribution of FC expenditures relative to FC allocations among specific FC infrastructure areas (Figure 1) did not differ substantially by accredited vs. not-accredited status.

Consistent with the findings of previous studies (9, 40, 42), we found accredited and not-accredited LHDs in the full dataset of 67 LHDs from the UCOA data to differ in some ways from each other. These 67 LHDs showed that, when compared to not-accredited LHDs, the accredited LHDs tended to have lead executives with graduate level degrees and served larger population sizes. The percent of accredited LHDs with a lead executive with an education level of a master's degree or higher was 87%, compared to 64% for LHDs in the UCOA sample that were not-accredited (Table 3). The percent of accredited LHDs serving population sizes >100,000 (population group 1, 2, and 3) in the sample was 70% compared to 50% for LHDs that were not accredited.

**Table 3:** Lead executive education levels and population size of LHDs that were accredited and those that were not from among LHDs in the UCOA data.

***N* = 67**	**Accredited**	**Not-accreditation**
**Executive education level**		
Master's or higher	87%	64%
Bachelor's or lower	13%	17%
Education level not reported	0%	19%
**Population group of LHD's jurisdiction**		
PopGrp1: >1 million	32%	3%
PopGrp2: (250,000–1 million)	19%	39%
PopGrp3: (100,000–249,999)	19%	8%
PopGrp4: <100,000	28%	50%

### Cluster Analysis: Distribution of FC Budget in Each FC Infrastructure Area (FC Allocation Pattern)

**Figure 2:**
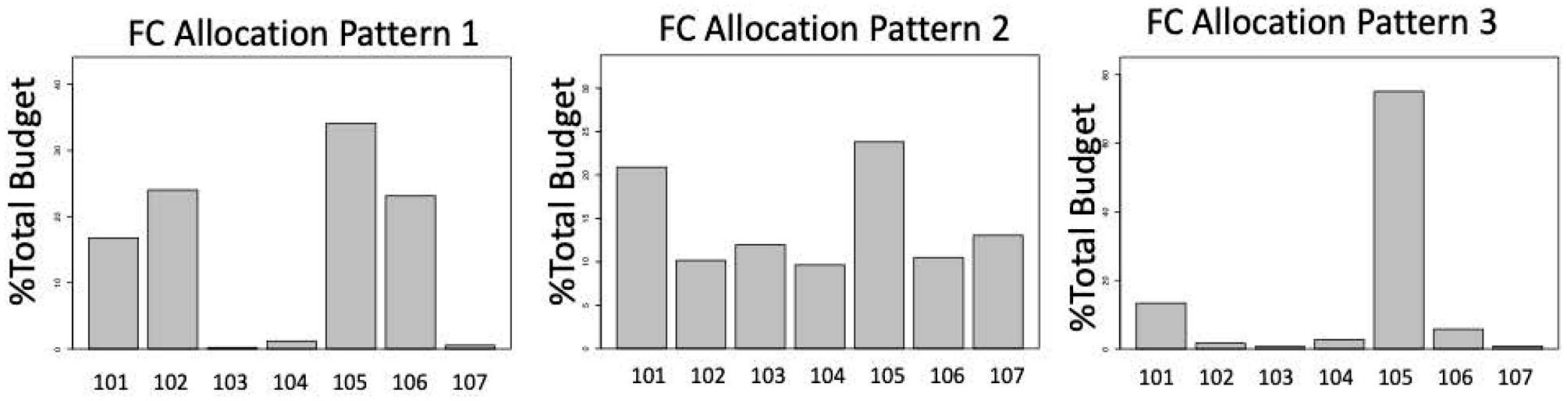
FC allocation patterns from cluster analysis of percent FC Budget on FC capabilities. Fcap101: Emergency preparedness and Response, fcap102: Assessment, fcap103: Communication, fcap104: Community Partnership and Development, fcap105: Organizational Competency, fcap106: Policy Development and Support, fcap107: Other Capabilities.

The result of the cluster analysis with the initial UCOA dataset (*n* = 67) showed three distinct clusters of LHDs with similar FC allocation patterns (percent of Total FC Budget spent on each FC infrastructure area). After including only accredited LHDs (*n* = 31) from the initial clusters, the percent of LHDs in each cluster remained very similar to the cluster for the initial 67 LHDs (Supplementary Appendix 2). The majority of the 31 LHDs (61%) fell into the Allocation Pattern 3 cluster. Figure 2 shows the average FC allocation patterns for LHDs in each cluster. LHDs in Allocation Pattern 1 allocated most of their FC budget to a mix of four FC areas (fcap101—Emergency preparedness and Response, fcap102—Assessment, fcap105—Organizational Competency, and fcap106—Policy Development and support) ranging from 18% to 30% of the Total FC Budget in each of these four areas. LHDs in Allocation Pattern 2 had more evenly distributed allocations, ranging from 10% to 23% in all seven FC programs. LHDs in Allocation Pattern 3 allocated more than 70% of their Total FC Budget to fcap105—Organization Competency.

### Total FC Budget Level and Performance Score in PHAB Accreditation Standards

Table 4 shows the change in performance score with respect to one dollar increase in Total FC Budget per capita. The aggregate performance score in PHAB accreditation standards was found to be associated with an increase in the Total FC Budget per capita. Aggregate performance score in PHAB accreditation standards increased by 0.2% with a unit increase in the Total FC Budget per capita. Most of the individual PHAB standards also showed positive associations between performance score improvements for each dollar increase in Total FC Budget–ranging from improvements of 0.08% to 1.2% on average. Negative coefficients were observed in five of the PHAB specific standards [Domain 1 Standard 1 (D1S1); Domain 1 Standard 3 (D1S3); Domain 1 Standard 4 (D1S4); Domain 5 Standard 3 (D5S3); and Domain 6 Standard 1 (D6S1); the description of these domains and standards are in Supplementary Appendix 1].

**Table 4:** Bayesian estimates for percent change (with 95% credible interval) in performance score with a unit change in FC budget per capita.

**Measure**	**Mean (%)**	**CI lower**	**CI upper**
Aggregate score	0.23	−0.09	0.56
D1S1	−0.48	−1.38	0.65
D1S2	0.53	−0.33	1.41
D1S3	−0.26	−1.20	0.71
D1S4	−0.90	−1.88	0.03
D2S1	0.85	−0.10	1.85
D2S2	1.22	−0.02	2.47
D2S3	0.40	−0.53	1.40
D2S4	0.24	−0.52	1.07
D3S1	0.25	−0.65	1.20
D3S2	0.08	−0.51	0.69
D5S1	0.14	−0.48	0.75
D5S2	0.28	−1.08	1.63
D5S3	−0.21	−1.20	0.76
D5S4	0.50	−0.82	1.86
D6S1	−0.52	−1.31	0.32
D6S2	0.22	−0.46	0.91
D6S3	0.77	−0.09	1.63
D9S1	0.89	−0.18	1.94
D9S2	0.44	−0.87	1.76

*D denotes PHAB domain, S denotes PHAB standards. Brief Domain description below. Full descriptions of domains and standards are described elsewhere and listed in **Supplementary Appendix 1** (31).*

*Domain Description: Domain 1—Conduct and Disseminate Assessments Focused on Population Health Status and Public Health Issues Facing the Community; Domain 2—Investigate Health Problems and Environmental Public Health Hazards to Protect the Community; Domain 3—Inform and Educate about Public Health Issues and Functions; Domain 5—Develop Public Health Policies and Plans; Domain 6—Enforce Public Health Laws; Domain 9—Evaluate and Continuously Improve Processes, Programs, and Interventions*.

### FC Allocation Pattern and Performance Score in PHAB Standards

To analyze the association between LHDs' performance score in PHAB accreditation standards and FC Allocation pattern, we used the FC Allocation cluster indices as the primary independent variable. Table 5 shows the percent difference in performance scores for FC Allocation Pattern 2 and Pattern 3, relative to Pattern 1. Positive values indicated improvements in performance scores relative to Pattern 1. Negative values indicated performance scores decreased relative to Pattern 1. On average, the results indicated an improvement of ~9% for an LHD with FC Allocation Pattern 2 (i.e., spending on a relatively evenly distributed pattern in all seven FC areas) relative to Pattern 1. The results also suggest a drop of ~8% in performance scores for Pattern 3 (i.e., spending primarily on Organization Competency) relative to Pattern 1. In terms of the relative improvement by cluster (Table 5), the best performance improvement was observed for FC Allocation Pattern 2 in most of the specific PHAB standards. However, FC Allocation Pattern 3 had the best performance in D2S2 and D6S3 (Supplementary Appendix 1).

**Table 5:** Bayesian estimates for percent change (with 95% credible interval) in performance score with change in FC Allocation Pattern 2 and Pattern 3 relative to Pattern 1.

**Measure**	**FC Allocation Pattern 2**			**FC Allocation Pattern 3**			**Relative improvement**
	**Mean**	**CI lower**	**CI upper**	**Mean**	**CI lower**	**CI upper**	
Average score	8.5	−13.5	34.9	−7.7	−23.6	11.3	C2 > C1 > C3
D1S1	−2.0	−29.6	33.8	−6.2	−26.9	20.5	C1 > C2 > C3
D1S2	6.2	−30.2	60.2	−11.0	−36.7	24.5	C2 > C1 > C3
D1S3	−14.5	– 42.5	30.5	−31.5	−50.5	−3.7	C1 > C2 > C3
D1S4	18.1	−17.6	68.3	−3.3	−27.0	28.5	C2 > C1 > C3
D2S1	−9.3	– 40.5	39.9	−6.8	−33.5	30.5	C1 > C3 > C2
D2S2	15.8	−28.9	83.6	22.0	−15.4	75.1	C3 > C2 > C1
D2S3	7.8	−31.3	68.6	−2.7	−33.5	40.8	C2 > C1 > C3
D2S4	16.1	−21.6	69.4	2.4	−25.7	39.2	C2 > C3 > C1
D3S1	7.8	−31.4	69.4	−24.4	−48.1	9.2	C2 > C1 > C3
D3S2	−14.6	−32.1	8.3	−3.5	−19.9	16.4	C1 > C3 > C2
D5S1	35.7	1.0	80.6	4.5	−16.7	32.9	C2 > C3 > C1
D5S2	17.5	−30.2	92.5	−15.2	−43.2	27.0	C2 > C1 > C3
D5S3	−19.8	−43.8	13.6	−21.4	−40.5	3.9	C1 > C2 > C3
D5S4	99.6	25.9	216.1	28.6	−10.3	86.1	C2 > C3 > C1
D6S1	6.2	−26.1	55.5	−12.1	−34.9	19.0	C2 > C1 > C3
D6S2	−4.6	−31.5	33.3	−13.5	−33.1	11.9	C1 > C2 > C3
D6S3	7.1	−28.2	61.4	10.1	−19.5	51.7	C3 > C2 > C1
D9S1	21.5	−24.2	94.8	−11.3	−39.2	30.0	C2 > C1 > C3
D9S2	10.7	−35.1	94.9	−21.2	−49.3	22.0	C2 > C1 > C3

***Domain Description**: Domain 1—Conduct and Disseminate Assessments Focused on Population Health Status and Public Health Issues Facing the Community; Domain 2—Investigate Health Problems and Environmental Public Health Hazards to Protect the Community; Domain 3—Inform and Educate about Public Health Issues and Functions; Domain 5—Develop Public Health Policies and Plans; Domain 6—Enforce Public Health Laws; Domain 9—Evaluate and Continuously Improve Processes, Programs, and Interventions. Full descriptions of domains and standards are described elsewhere and listed in Supplementary Appendix 1 (31)*.

## Discussion

No known prior study has investigated LHD performance score in PHAB accreditation standards with respect to specific FC expenditures. This study supports the notion that LHDs with adequate resources are more likely to be successful in accreditation, particularly when considering FC expenditures, given the positive association found between performance score in PHAB accreditation and the Total FC Budget. The analyses suggest that one dollar increase in FC spending per capita could improve LHDs' performance score in PHAB accreditation standards by 0.2% on average. This overall improvement is a result of positive associations in most of the individual PHAB standards. While previous studies have shown that accredited LHDs tend to spend more on PH activities than those that are not-accredited (9), the results of this analysis further extends our understanding of PH system infrastructure and the importance of adequate resources for foundational capabilities for LHDs to perform well in PHAB accreditation assessments. Lack of adequate resources in these areas could put LHDs at a disadvantage during preparation and assessment for accreditation (47, 48).

Public health leaders continue to advocate for more resources to improve LHDs' core capabilities necessary to respond effectively, on a day-to-day basis and during public health crises (5, 49–51), since LHD core capabilities are assumed to relate to improvements in the health of the public. The COVID-19 pandemic further reinforced the need for better funding to improve PH infrastructure and preparedness in the United States. For example, in 2021 U.S. Senator Patty Murray of Washington State, introduced a legislative effort advocating for more funding to improve the PH infrastructure in an amount that would reach $4.5 billion annually in 2025 (17). The legislation not only called for funding to revamp the infrastructure of LHDs but also called for “a standardized approach to financial reporting” (such as the UCOA) and funding to develop and implement an accreditation program to ensure LHDs are meeting rigorous standards (17). Since FC are considered the building blocks of LHDs' activities to provide PH services, providing adequate funding for tracking, supporting, and allocating FC could improve agency performance in delivering PH essential services and be better prepared for PH crises, which in turn can produce better community health outcomes (9, 16, 24).

While it is apparent that increasing funding for PH infrastructure may lead to improved performance for LHDs in the United States (52), and consequently improved performance in PHAB accreditation scores, this study also suggests that the approach to which LHDs allocate available funds to the various FC areas could impact their performance scores in PHAB accreditation standards. The majority of LHDs in the sample examined allocate a large percentage of their FC budget to Organizational Competency (cluster 3—Allocation Pattern 3). This spending pattern appears not to produce optimal performance in PHAB accreditation standards, compared to LHDs that allocate resources more evenly across FC areas, as a more mixed spending pattern (Allocation Pattern 2) showed the best performance in this study. Recent research on the rate of accreditation uptake among small LHDs indicates that LHDs with a more balanced mix of activities were more likely to participate in accreditation (53, 54). Here, additional knowledge is gained regarding how LHDs can prioritize their FC programs to achieve accreditation. PHAB could also encourage the allocation of resources across a mix of FC areas as a means to support accreditation success.

As LHDs advocate for more resources and look for ways to structure limited resources for better community health outcomes, there is a need to embrace evidence-based approaches when setting priorities (55–59). In a study by Baum et al. (58), how LHD leaders make funding allocation decisions, the process they used to make decisions, and the factors that influence decisions on how public health resources are used to ensure effective activities and access to needed services were examined. The study showed that only a small number of LHDs used evidence-based approaches such as economic analyses or conducted needs assessments when setting priorities. Instead, most agencies based their decisions on previous budget allocations and input from their boards of health to make allocation decisions (58).

Public health leaders' and policy makers' emphasis on addressing the issue of limited resources for LHDs should also include evidence-based financial models to ensure PH resources are efficiently managed for optimal health outcomes (17, 59). Therefore, effective resource allocation strategies are needed for LHDs to achieve optimal performance in delivering their PH programs and services. Understanding FC allocation patterns in relation to performance score in PHAB standards provides information and potential guidance on how FC resource allocation may affect PH agencies' performance.

The findings of this study also reinforce the convergence of the goals of the FPHS framework and PHAB accreditation and demonstrate the potential of PHAB accreditation standards as an effective instrument for assessing performance in PH systems. The alignment of PHAB standards with the FPHS framework enable a means to gain further insight here into the significance of FC expenditures in relation to PH performance and quality improvement. Within the FPHS framework, FC encompass LHDs' structure and processes, and thus represent a component of the PH system from which performance information could be obtained. Maintaining FC, in this case synonymous with increases in FC expenditures, would appear to enhance PH services that promote healthy communities. Finally, the use of standardized financial data along with PHAB accreditation data demonstrates the value of uniform, structured PH information for comparative financial and performance assessment.

### Limitations

Due to our small sample size, the predictive power and the number and range of covariates in the model were limited. Thus, we refrained from generalizing the results to all LHDs present in the United States. However, the use of Bayesian estimation methods was effective for handling sample size limitations. Also, descriptive analyses we performed suggest the sample (*n* = 31) was, nonetheless, generally representative of the 67 LHDs present in the UCOA data. The same was true with the 31 accredited LHDs vs. the 250 accredited LHDs in the PHAB data, thus, our sample size represented the diversity in the pool of our dataset. However, we recognize this as a study limitation because the 250 LHDs that were accredited by PHAB at the time of this analysis are <10% of all LHDs and thus may not represent all LHDs. In addition, the ordinal to numerical transformation methods used could be considered a potential limitation. Furthermore, this analysis does not assume causal inference, and the potential feedback loop that may exist between total FC budget and PHAB scores was not considered in this analysis. Future research with longitudinal data should explore this interaction.

## Conclusion

Findings of this study suggest that investments in FC could improve LHD performance score in PHAB accreditation standards and support their ability to meet the required capabilities for improving community health outcomes. The study also suggests that allocating available FC resources across the various FC programs could support better LHD performance, as indicated by accreditation standard scores. Insights gained regarding FC allocation patterns and their impact on LHD performance scores could help LHDs objectively allocate their resources based on the specific purpose or target they want to achieve at a particular period. LHDs are generally underfunded (8, 60) and managing available limited resources for optimal performance is key (58, 61). Understanding how public health investments in FC improve LHDs' PHAB accreditation performance score will extend our knowledge of how PH resources and capabilities relate to PHAB accreditation performance and could also inform how LHDs can improve their budgeting and resource allocation for better performance.

## Data Availability Statement

The data analyzed in this study is subject to the following licenses/restrictions. Data used in the research are not publicly available, but they can be requested from the institutions that provided the data. Requests to access these datasets should be directed at: Jessica Kronstadt, jkronstadt@phaboard.org, and UW PHAST UCOA, phast@uw.edu.

## Author Contributions

All authors listed have made a substantial, direct, and intellectual contribution to the work and approved it for publication.

## Funding

Funding for this study was provided in part by the Robert Wood Johnson Foundation (Grant #75301). Open Access funding was provided by the Ellery and Kirby Kramer Endowed Professorship in Nursing held by BB.

## Conflict of Interest

The authors declare that the research was conducted in the absence of any commercial or financial relationships that could be construed as a potential conflict of interest.

## Publisher's Note

All claims expressed in this article are solely those of the authors and do not necessarily represent those of their affiliated organizations, or those of the publisher, the editors and the reviewers. Any product that may be evaluated in this article, or claim that may be made by its manufacturer, is not guaranteed or endorsed by the publisher.
