# Supplementary material for: Associations Between Local Health Department Expenditures on Foundational Capabilities and PHAB Accreditation Standards Scores
Source: Front Public Health. 2022 May 25;10:861587. doi: 10.3389/fpubh.2022.861587 (PMC9174657; doi:10.3389/fpubh.2022.861587)
Supplement: Supplementary file 1 [file Table_1.DOCX]

## Appendix 1

Selected (19) Standards and FC areas alignment: D – Domain, S – Standards. Detailed description of the alignment is available elsewhere (1).

<https://phaboard.org/wp-content/uploads/PHABSM_WEB_LR1-1.pdf> (2)

| Domain | Standards | Domain/Standard  Composite | FC Alignment |
| --- | --- | --- | --- |
| **1:** Conduct and disseminate assessments focused on population health status and public health  issues facing the community | 1 | D1S1 | Assessment/Surveillance;  Health Equity;  Information Technology Services, including Privacy and Security |
|  | 2 | D1S2 |  |
|  | 3 | D1S3 |  |
|  | 4 | D1S4 |  |
| **2:** Investigate health problems and environmental public health hazards to protect the community | 1 | D2S1 | Emergency Preparedness and Response;  Communications; |
|  | 2 | D2S2 |  |
|  | 3 | D2S3 |  |
|  | 4 | D2S4 |  |
| **3:** Inform and educate about public health issues and functions | 1 | D3S1 | Leadership;  Health Equity;  Communication |
|  | 2 | D3S2 |  |
| **5:** Develop public health policies and plans | 1 | D5S1 | Policy Development and Support;  Assessment/Surveillance;  Emergency Preparedness and Response;  Community Partnership Development;  Leadership and Governance |
|  | 2 | D5S2 |  |
|  | 3 | D5S3 |  |
|  | 4 | D5S4 |  |
| **6:** Enforce public health laws | 1 | D6S1 | Emergency Preparedness and Response;  Policy Development and Support;  Legal Services and Analysis |
|  | 2 | D6S2 |  |
|  | 3 | D6S3 |  |
| **9:** Evaluate and continuously improve processes, programs, and interventions | 1 | D9S1 | Quality Improvement |
|  | 2 | D9S2 |  |

**APPENDIX 2**

| **Table A2**: Comparison of LHDs from full UCOA dataset and accredited sample by FC expenditure allocation pattern derived from cluster analysis of percent FC budget expenditure in each FC infrastructure area. | | | | |
| --- | --- | --- | --- | --- |
| Cluster # | LHDs in full UCOA dataset  (n = 67) | | Regression Model LHD Sample (n = 31)  (Accredited LHDs in UCOA data) | |
| 1 | 12 | 18% | 6 | 19.4% |
| 2 | 14 | 21% | 6 | 19.4% |
| 3 | 41 | 61% | 19 | 61.2% |
| Total | 67 | 100% | 31 | 100% |

**References**

1. Public Health National Center for Innovations (PHNCI). Aligning Accreditation and the Foundational Public Health Capabilities: Public Health National Center for Innovations; 2018 [Available from: <https://phnci.org/uploads/resource-files/Aligning-Accreditation-and-the-Foundational-Public-Health-Capabilities-November-2018.pdf> (Accessed March 18, 2020).

2. Public Health Accreditation Board (PHAB). Standards and Measures Version 1.5 2013 [Available from: <https://phaboard.org/wp-content/uploads/PHABSM_WEB_LR1-1.pdf>. (Accessed March 15, 2020).
